# Asymmetric electrode incorporated 2D GeSe for self-biased and efficient photodetection

**DOI:** 10.1038/s41598-020-66263-8

**Published:** 2020-06-10

**Authors:** Muhammad Hussain, Sikandar Aftab, Syed Hassan Abbas Jaffery, Asif Ali, Sajjad Hussain, Dinh Nguyen Cong, Raheel Akhtar, Yongho Seo, Jonghwa Eom, Praveen Gautam, Hwayong Noh, Jongwan Jung

**Affiliations:** 1 Department of Nanotechnology and Advanced Materials Engineering, and HMC, Sejong University, Seoul, 05006 South Korea; 2 Department of Physics & Astronomy and Graphene Research Institute-Texas Photonics Center International Research Center (GRI–TPC IRC), Sejong University, Seoul, 05006 Korea; 3 Department of Electrical Engineering University of Lahore, Islamabad, Pakistan

**Keywords:** Materials science, Nanoscience and technology

## Abstract

2D layered germanium selenide (GeSe) with *p*-type conductivity is incorporated with asymmetric contact electrode of chromium/Gold (Cr/Au) and Palladium/Gold (Pd/Au) to design a self-biased, high speed and an efficient photodetector. The photoresponse under photovoltaic effect is investigated for the wavelengths of light (i.e. ~220, ~530 and ~850 nm). The device exhibited promising figures of merit required for efficient photodetection, specifically the Schottky barrier diode is highly sensitive to NIR light irradiation at zero voltage with good reproducibility, which is promising for the emergency application of fire detection and night vision. The high responsivity, detectivity, normalized photocurrent to dark current ratio (NPDR), noise equivalent power (NEP) and response time for illumination of light (~850 nm) are calculated to be 280 mA/W, 4.1 × 10^9^ Jones, 3 × 10^7^ W^−1^, 9.1 × 10^−12^ WHz^−1/2^ and 69 ms respectively. The obtained results suggested that p-GeSe is a novel candidate for SBD optoelectronics-based technologies.

## Introduction

Two-dimensional (2D) materials have chronically been the most widely studied materials, particularly after the successful scotch tape test to exfoliate graphene by Andre Geim and Kostya Novoselov in 2004^1^. 2D materials possess excellent electrical and mechanical properties toward diverse electronic device applications. Graphene, being the prototype of 2D materials^2,3^, has been studied broadly for its exotic electrical, optical, and mechanical properties^3,4^. Besides, the group-*IV* transition metal dichalcogenides (TMDs) having a bandgap of around 1 to 2 eV^5–7^ have attracted increasing interest because of their promising electronic and optoelectronic device applications^3,8–21^. Graphene possesses extremely high carrier mobility (>10^5^ cm^2^ V^−1^ s^−1^), but the absence of band gap limits its electronic and optoelectronic applications^22^. Therefore, TMDs with the properties of graphene-like stature, bandgap tunability, weak van der Waals-like forces and stability have intrigued the interest of the scientific community. TMDs are the family of 2D materials having the chemical composition of MX_2_, where M stands for the transition metal elements (M = Mo, W, Ta, Ge…etc) and X for the chalcogen elements (X = Se, S and Te). Among TMDs, Ge-based materials are preferred for applications due to their abundance on earth and environmentally friendly nature^23^. With Se, the *p*-type Germanium from a narrow bandgap semiconductor material as *p*-GeSe having exciting application in near-infrared (NIR) photodetectors and electron tunnelling devices. *p*-GeSe has an indirect bandgap of 1.08 eV in the bulk^24,25^, and a direct bandgap of ~1.7 eV in monolayers^24,26,27^. Few layers of *p*-GeSe can be obtained from bulk by mechanical exfoliation method^28^. Among the many applications, p-GeSe shows tremendous capability in the realm of photovoltaics, because of its excellent optical, material and electrical properties. Therefore, it is well known as substitution of phosphorene^29^. Moreover, GeSe is considered as an ambient stable material ^30,31^. Single crystal *p*-GeSe has hole mobilities of 95 cm^2^ V^−1^ s^−1^ at 300 K and 663 cm^2^ V^−1^ s^−1^at 112 K^24,32^. Theoretical elucidations predicted that the average hole mobility for monolayer GeSe is as high as 1.1 × 10^3^ cm^2^ V^−1^ s^−1^at 300 K^24^. Meanwhile, Metal/TMD interface plays an active role in enhancing photoresponse in photodetection applications^33–35^. Therefore, it is important to understand the nature of the Metal/TMD interface in device applications. Recently, 2D heterostructure has been extensively studied for an ideal platform for diode devices, e.g., GaSe/MoSe_2_^36^, MoS_2_/Black Phosphorus^37^, MoS_2_/p-Si junction^38^, WSe_2_/MoS_2_ heterojunction^39^ and p-type MoSe_2_ via Nb-elemental doping^40^ etc. Despite thorough research on bulk GeSe crystals and numerous theoretical calculations on monolayers^24,26^,^41^, to the best of our knowledge, by using p-GeSe on (Cr/Au) and (Pd/Au) contact, no experimental investigations have focused on Schottky-Barrier diode (SBD) by asymmetric metal contact with NIR wavelength excitation.

The metal-semiconductor (MS) junction is well known to be a Schottky barrier or ohmic junction based on the corresponding work function of metal compared to the semiconductor and its conductivity type. For the p-type semiconductor, the Schottky barrier is formed when metal work function is smaller than the semiconductor^5,42–44^. SBD formed by Schottky barrier is a widely used component in electronics such as multipliers, microwave mixer and photodetectors, due to its high-frequency capability^45^ and strong nonlinear current-voltage characteristics. Moreover, it is known that SBDs show promising electronic applications and can be used extensively in power electronics due to the low voltage drop in forward bias. It can also be used as photodiodes, power diodes, sensors, varistors and varactors owing to the nonlinear *I-V* behavior^5,46^.

In this research work, the p-GeSe based FETs were fabricated by using two metals contacts (Cr/Au) and (Pd/Au) to determine the charge carrier transport and the interface characteristics at the junction. The hole mobility of p-GeSe with Pd/Au electrodes was ~5.0 cm^2^V^−1^s^−1^. Notably, *p*-GeSe is in ohmic contact with (Pd/Au) while having a Schottky barrier with (Cr/Au). SBD was made from asymmetric electrodes of (Pd/Au) and (Cr/Au). We found the rectifying behavior with an on/off ratio of ~10^3^. Besides, we also have extensive studies of the photoconductivity of all fabricated devices under different laser illumination with a wavelength from 220 nm to 850 nm. It is observed that the SBD is highly sensitive to NIR light irradiation at zero voltage with good reproducibility. The responsivity, detectivity, normalized photocurrent to dark current ratio (NPDR), noise equivalent power (NEP) and response time are estimated to be 220 mA/W, 4.1 × 10^9^ Jones, 3 × 10^7^ W^−1^, 9.1 × 10^−12^ WHz^1/2^ and 69 ms, respectively. There were a few reports in self-powered SBD using 2D materials such as MoS_2_, WS_2_, InSe, and BN,but NIR detection using 2D GeSe has not reported yet. The obtained results suggest that p-GeSe is a novel candidate for SBD optoelectronics-based technologies.

## Results and Discussion

**Figure 1:**
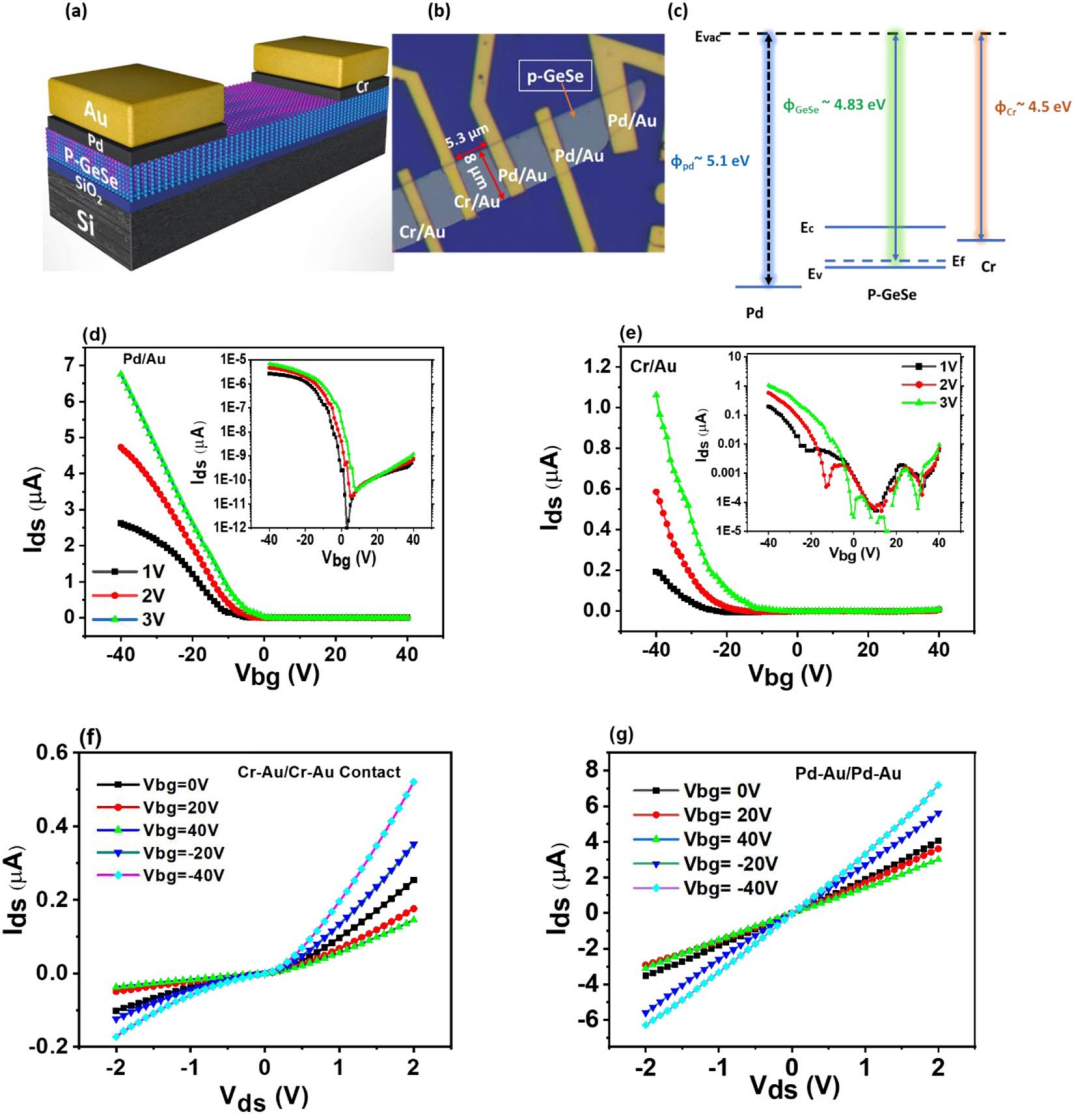
(**a**) Schematic view of p-GeSe Schottky diode. (**b**) Optical microscope image of the device. (**c**) Band diagram of Pd**/**p-GeSe**/**Cr. *I*_*D*_*-V*_*G*_ characteristics of (**d**) p-GeSe FET with Pd/Au and (**e**) Cr/Au with V_ds_ = 1 V to 3 V.T.the semi-logarithmic plots of transfer characteristics are depicted in the insets on each figure. *I*_*D*_*-V*_*D*_ characteristics as a function of gate bias (**f**) Schottky contacts effect of p-GeSe with Cr/Au. (**g**) Ohmic contact behavior with Pd/Au.

Figure 1a illustrates the schematic diagram of the as-fabricated device having both Schottky and ohmic junction due to asymmetric contact electrodes (Pd/Au and Cr/Au) with a p-GeSe on SiO_2_/Si substrate. The optical microscope image of the device is illustrated in Fig. 1b. The detail transfer flakes process is provided in experimental detail. Atomic force microscopy (AFM) was used to evaluate the thickness of p-GeSe (~40 nm) presented in Fig. S1. Raman spectrum of GeSe nanoflake was depicted in Fig. S1. It has been stated that the metals having lower work function are suitable for p-GeSe to form a facile Schottky barrier height^5,43,44^. It was found that p-GeSe and metal interface could be either an ohmic or rectifying behavior depending on metals and semiconductor working function value. Figure 1c depicts GeSe (Φ ~ 4.83 eV)^47^,Pd (Φ ~ 5.6 eV)^43^ and Cr (Φ ~ 4.5 eV)^5,48^ were used as electrode contacts in GeSe flake. Initially, the electrical characteristics of the p-GeSe FETs with Cr/Au and Pd/Au electrodes were measured. The transfer characteristics show typical p-type semiconductor characteristics, depicted in Fig. 1(d,e). The field-effect carrier mobility (${{\rm{\mu }}}_{{\rm{FE}}}$) was calculated for individual metal contacts using the following equation:

1 $${{\rm{\mu }}}_{FE}=\frac{{\rm{L}}}{{\rm{W}}}(\frac{{{\rm{dI}}}_{{\rm{ds}}}}{{{\rm{dV}}}_{{\rm{bg}}}})\frac{1}{{{\rm{C}}}_{{\rm{bg}}}{{\rm{V}}}_{{\rm{ds}}}}$$

where W and L are the channel width and length, respectively, C_bg_ is the gate capacitance (~115 aF/µm^2^) for the 300 nm-thick SiO_2_/Si substrates and $(\frac{{{\rm{dI}}}_{{\rm{ds}}}}{{{\rm{dV}}}_{{\rm{bg}}}})$ is the slope of the transfer curve. The hole mobility of GeSe with Cr/Au contacts was measured to be 1.98 cm^2^V^−1^s^−1^ with a current on/off ratio of ~10^2^, while the mobility of GeSe with Pd/Au electrodes was estimated to be 5.02 cm^2^V^−1^s^−1^ with ~10^4^. Our findings revealed that the mobility of GeSe with Cr/Au decreased than that of one with Pd/Au because of the Schottky junction formed at both Cr/Au contacts, which resist to the follow of charges. In the Pd/Au configuration, both contacts were formed with ohmic behavior because of the high work function of Pd (Ф~ 5.6 eV)^49^ further, the gate voltage (V_bg_) dependent I_D_-V_D_ output characteristics shows rectification behavior for the Cr/Au metal contacts because of the formation of the Schottky barrier with P-GeSe as depicted in Fig. 1f. From the linear characteristics of *I-V* output, we could predict that ohmic junction was formed with Pd/Au contacts, as shown in Fig. 1g.

**Figure 2:**
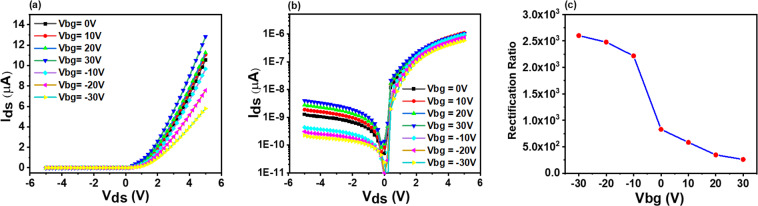
(**a**) *I-V* characteristics *of* p-GeSe Schottky junction between Cr/Au-Pd/Au contacts at different gate voltages. (**b**) the semi-logarithmic plots of output characteristics (**c**) Gate dependent rectification ratio of p-GeSe Schottky junction.

The rectification of p-GeSe with asymmetric contacts of Pd/Au and Cr/Au explicitly showed gate-dependent rectification, especially high rectifying behavior at a negative gate voltage due to higher Schottky barrier height between Cr and GeSe, as illustrated in V. When the forward bias voltage is applied, the rectifying current increases because of decreased barrier height. A rectification ratio (defined as the ratio of reverse current I_r_ and forward current I_f_) is obtained 10^3^ as depicted in Fig. 2c, which is consistent with the other devices shown in Fig. S3.

We estimated the ideality factor to confirm the good performance of rectifying behavior of SBD by using thermionic emission theory. According to this theory, the current I_D_ through a metal-semiconductor junction is given by^50,51^

2 $${I}_{D}={I}_{S}\left[\exp \left(\frac{qV}{n{K}_{B}T}\right)-1\right]$$

where ${I}_{S}$ is the reverse bias saturation current, $n$ is an ideality factor, q is an elementary charge, T is the temperature, and ${K}_{B}$ is the Boltzmann’s constant. After interpretation above equation becomes

3 $$\mathrm{ln}({I}_{D})=\,\mathrm{ln}({I}_{S})+\left(\frac{q}{n{K}_{B}T}\right)V$$

The ideality factor (n) can be obtained via the following equation:

4 $$n=\left(\frac{q}{{K}_{B}T}\right)(\frac{{\rm{d}}V}{{\rm{dln}}{I}_{D}})$$

The lowest value of the ideality factor (n) we evaluated was ~1.8. To extract the Schottky barrier height (SBH) of Pd (ϕ = 5.6 eV) and Cr (ϕ = 4.5 eV), respectively, we measured temperature-dependent transfer characteristics as shown in Fig. S2.

**Figure 3:**
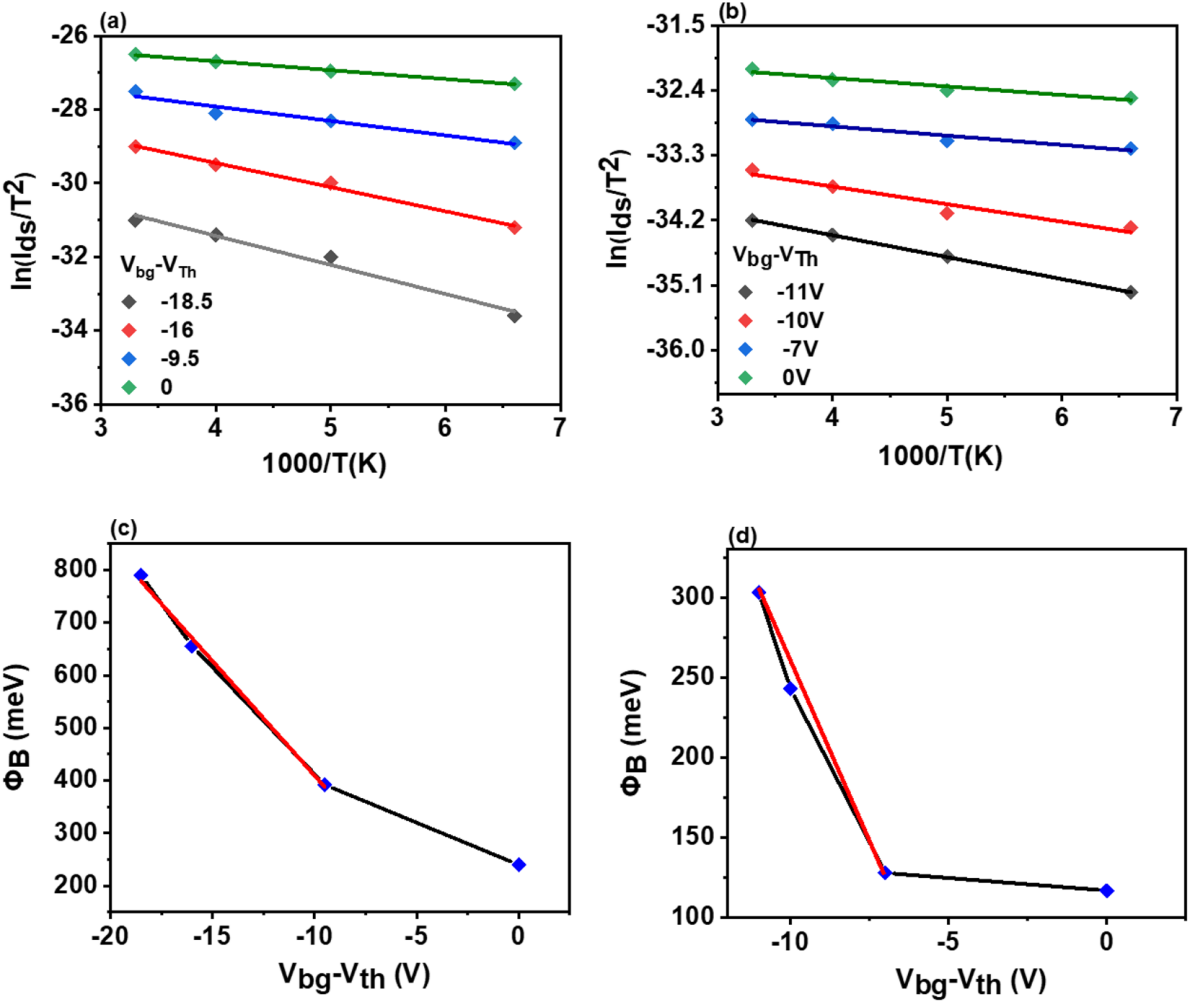
(**a**) Typical Richardson’s plot ln (I_ds_/T^2^) versus 1000/T for (**a**) Cr/Au and (**b**) for Pd/Au metal contacts. Corresponding Schottky barrier Potential (**c**) at the p-GeSe/ Cr/Au and (**d**) Pd/Au junction as a function of Vbg-Vth.

According to thermionic emission theory, we used the following equation^5,52–54^

5 $${{\rm{I}}}_{{\rm{ds}}}={A}_{device}{A}^{\ast }\,{{\rm{T}}}^{2}\exp \left[-\frac{{\rm{q}}}{{{\rm{k}}}_{{\rm{B}}}{\rm{T}}}\,\left({\Phi }_{{\rm{B}}}-\frac{{{\rm{V}}}_{{\rm{ds}}}}{{\rm{n}}}\right)\right]$$

where ${A}_{device},\,{A}^{\ast }$ are the area of the detector and Richardson constant, q is the elementary charge of the electron, T is the temperature K_B_ is Boltzmann’s constant, V_ds_ the source-drain voltage, and n is the ideality factor. We drew the Arrhenius plot of the device at different gate voltages for both metals Pd and Cr shown in Fig. 3a,b, respectively. We estimated the SBH for Pd and Cr with p-GeSe by taking the slope of ln (I_ds_/T^2^) versus 1000/T, as shown in Fig. 3c,d. We found the SBH of Cr with p-GeSe is much higher than SBH of Pd because of ohmic behavior between Pd and p-GeSe.

Moreover, we also extensively studied self-powered photoresponse characteristics of SBD. Self-power photodetectors are devices in which photogenerated charge carriers are separated by the built-in potential and there is no requirement of any external power source. Pd/Au-GeSe-Pd/Au structure does not show any photoresponse at zero bias since it has both ohmic contacts. However, Cr/Au-GeSe-Cr/Au form Schottky contacts, which may show a photoresponse at zero bias. But the symmetric contact electrodes may cause a net null current due to equal but opposite directions of photogenerated carriers. Hence, we designed a dual junction photodetector with asymmetric contacts electrodes having a configuration of Pd/Au-GeSe-Cr/Au. This photodetector uses the built-in potential present in metal contact and p-GeSe junction interface (forming Schottky junction) to separate and fast transfer of photogenerated electron-hole pairs, which empowers them to detect light signals without an external bias voltage.

**Figure 4:**
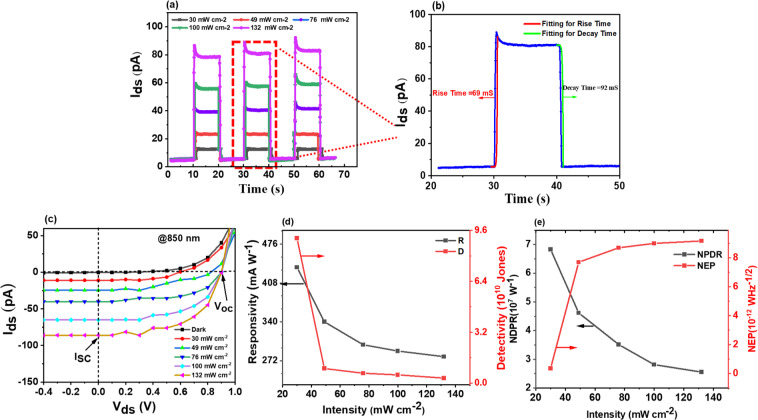
(**a**) Time dependent photoresponse of p-GeSe Schottky junction under illuminations with different laser light (@850 nm) intensity at V_ds_ = 0 V. (**b**) rise time and decay time (**c**) *I-V* characteristics *of* p-GeSe Schottky junction under dark and variable intensities (**d**) responsivity, R (mA W^−1^), and detectivity, D (Jones) with the variation of light intensities (**e**) normalized photocurrent to dark current ratio NPDR (W^−1^) and noise equivalent power NEP (W Hz^−1/2^) with varying light intensities.

Figure S4 illustrates the wavelength-dependent photovoltaic behavior under irradiation of light with wavelength (λ) of 220 nm, 530 nm, and 850 nm at zero voltage bias in p-GeSe Schottky diode, as illumination wavelength decreased, the reverse and forward bias currents drastically increased. This implies that photoexcited electron-hole pairs significantly increases with greater photon energy^55–57^. Figure S4a shows the output characteristics of the device under the illumination of light having wavelengths (λ) 220 nm, 530 nm and 850 nm, respectively, and Fig. S4(b–d) depicts the transient dynamic photoresponse. Figure 4c shows the *I-V* characteristics of the device under the illumination of near-infrared radiation (NIR) with a wavelength of 850 nm. The linear plot explicitly shows that in the presence of photogenerated current at the zero-bias device can be a self-powered photodetector under the action of open-circuit voltage (V_oc_). Furthermore, with irradiation of various intensities ranging from 30 to 132 mW cm^−2^, the device clearly shows systematic deviation from the dark level. We observed open-circuit voltage (V_oc_) of 0.9 V and short-circuit current (I_sc_) of 80 pA for the 132 mW cm^−2^ light intensity. The highest values of V_oc_ and I_sc_ were obtained with a power intensity of 132 mW cm^−2^ due to excessive photogenerated charge carriers under the effect of abundant photons in the high intensity of light.

The transient photoresponse is a very critical parameter for a photodetector to be used in emergency applications such as fire detection, night vision, etc. Therefore, the dynamic photoresponse rise and fall time of the p-GeSe SBD was observed under NIR laser light irradiation with a wavelength (λ) 850 nm. The rise time (τ_r_, time taken by device to reach 90% from 10%) and fall time (τ_f_, time taken by device to decay from 90% to 10%)^58^ of the p-GeSe Schottky barrier diode shows values of 69 and 92 ms, respectively (shown in Fig. 4b).

To further validate the gate tunable self-powered photovoltaic effect, we characterized the time-dependent photoresponse at various gate voltages. We realized that, without an external bias voltage and illuminating environment, the device reached its thermal equilibrium state when the Fermi levels of both metal and GeSe are aligned, resulting in the formation of a Schottky barrier (ohmic behavior) at Cr (Pd) and GeSe interface. When applying a back-gate voltage of Vg > 0, the electrons are attracted to the interface between p-GeSe and SiO2 to form accumulation layer, the Fermi level of p-GeSe shifts upward that attributed to electrostatic doping of electrons, results in a larger potential hole barrier between the p-GeSe and Cr. Consequently, a built-in potential at Cr-GeSe interface increased, this selective barrier at the junction is the means of separating charges during electron-holes generation under illumination^18,59^. It is the key to the production of a photovoltaic electric current^60–62^. Therefore, upon illumination of NIR wavelength of 850 nm with a power of 76 mWcm^−2^ yielding the greater photovoltaic current with an increase of *Vg* from 0 V to 40 V without external power. In contrast, at *Vg* < 0 the Fermi level of p-GeSe moves downward, yielding smaller the hole potential barrier, results in smaller photovoltaic current under the illumination of the light of the same condition, shown in Fig. S5(a). Additionally, to investigate the stability of the photovoltaic performance of the device, we measured two more p-GeSe Schottky photodetector devices. All devices showed same reproducibility which is consistent with previous device measured results shown in Fig. S5(c).

The other vital figures of merit, for instance, responsivity, detectivity, normalized photocurrent to dark current ratio, and noise equivalent power are calculated with the variation of light power intensities. The responsivity (${\rm{R}}={J}_{p}/{P}_{in}$, where J_p_ is photocurrent density and P_in_ is input power per area) and detectivity ($D=R/\sqrt{{2J}_{d}}$, where q is elementary charge and J_d_ is dark current density) are important aspects of photodetector^63–65^ shown in Fig. 4d. The greater value of responsivity is caused by the higher photocurrent^58,63^. Similarly, the device having a lower dark current provides the higher detectivity. Thus, the greater values of both R and D are an important feature of an efficient photodetector^67,58,66^. Our photodetector, based on p-GeSe SBD, showed a high responsivity of 280 mAW^−1^ and a detectivity of 4.1 × 10^9^ jones with a power intensity of 132 mW cm^−2^. Figure 4e shows the normalized photocurrent to dark current ratio (NPDR = R/I_d_, where R is responsivity and I_d_ is the dark current) and noise equivalent power (NEP = 1/(${\rm{NPDR}}\sqrt{2q/{I}_{d}}$)). The NPDR of 3 × 10^7^w^−1^ and NEP of 9.1 × 10^−12^ WHz^−1/2^ were investigated at a power intensity of 132 mW cm^−2^. The NEP revealed that the photodetector based on p-GeSe Schottky barrier diode is capable of detecting power as low as picowatt, which is very crucial for emergency applications.

## Conclusion

A self-powered photodetector was designed using asymmetrical metals contacts on a 2D semiconductor (p-GeSe). It has demonstrated outstanding gate dependent rectifying behavior with an excellent on/off ratio (up to 10^3^). In addition, stable rectifying behavior was also obtained with a built-in potential at the junction. This interfacial potential governs the separation of the photogenerated charge carriers making the fabricated device self-powered for optoelectronics applications. It has also exhibited a high rise and fall time of 69 and 92 ms, respectively. Moreover, promising figures of merits were obtained, such as responsivity, detectivity, normalize photocurrent to dark current ratio and noise equivalent power of 280 mAW^−1^, of 4.1 × 10^9^ Jones, 3 × 10^7^W^−1^ and 9.1 × 10^−12^ WHz^−1/2^ respectively. The NEP revealed that the photodetector based on p-GeSe Schottky barrier diode is capable of detecting power as low as picowatt, which is very crucial for emergency applications.

## Methods

Atomically thin flakes of p-GeSe were peeled from their parent bulk crystals using a scotch tape mechanical exfoliation technique, similar to that employed for the exfoliation of graphene^2,23^ and transferred onto Si/SiO_2_ (300 nm) substrate using transparent poly (dimethyl siloxane) (PDMS) stamp by aligned dry transfer^24,68^. Multilayer p-GeSe nanoflakes were identified by optical microscope, nanoflakes and were characterized by Raman spectroscopy and the thickness of p-GeSe (~40 nm) approximately equal to 40 layers evaluated by using atomic force microscopy (AFM). Electrodes were printed using electron beam lithography and Pd/Au (10/20 nm) and Cr/Au (10/20 nm) was evaporated and liftoff were carried out on multilayer p-GeSe nanoflakes. The electrical transport measurements at room temperature were characterized by using Keithley 4200A-SCS Parameter Analyzer. The photoresponse of SBD photodetector was measured using a continuous wave laser beam from the diode laser (from 220 nm to 850 nm) directly illuminated onto the device.

## Supplementary information

Supplementary information.
