# Supplementary material for: Asymmetric electrode incorporated 2D GeSe for self-biased and efficient photodetection
Source: Sci Rep. 2020 Jun 10;10:9374. doi: 10.1038/s41598-020-66263-8 (PMC7286883; doi:10.1038/s41598-020-66263-8)
Supplement: Supplementary file 1 — Supplementary information. [file 41598_2020_66263_MOESM1_ESM.docx]

# Asymmetric electrode incorporated 2D GeSe for self-biased and efficient photodetection

# Muhammad Hussain^1^, Sikandar Aftab^2^, Syed Hassan Abbas Jaffery^1^, Asif Ali^1^, Sajjad Hussain^1^, Dinh Nguyen Cong^1^, Raheel Akhtar^3^, Yongho Seo^1^, Jonghwa Eom^2^, Praveen Gautam^2^, Hwayong Noh^2^ & Jongwan Jung^1*^

^1^Department of Nanotechnology and Advanced Materials Engineering, and HMC, Sejong University, 05006, South Korea.

^2^Department of Physics & Astronomy and Graphene Research Institute-Texas Photonics Center International Research Center (GRI–TPC IRC), Sejong University, Seoul 05006, Korea.

^3^Department of Electrical Engineering University of Lahore, Islamabad.

^*^jwjung@sejong.ac.kr

**Figure S1.** (a) Raman spectra of p-GeSe flakes. (b) AFM image of p-GeSe Schottky junction on SiO_2_/ Si substrate. (c) height profile from the AFM, the thickness of p-GeSe flake was ~40 nm.


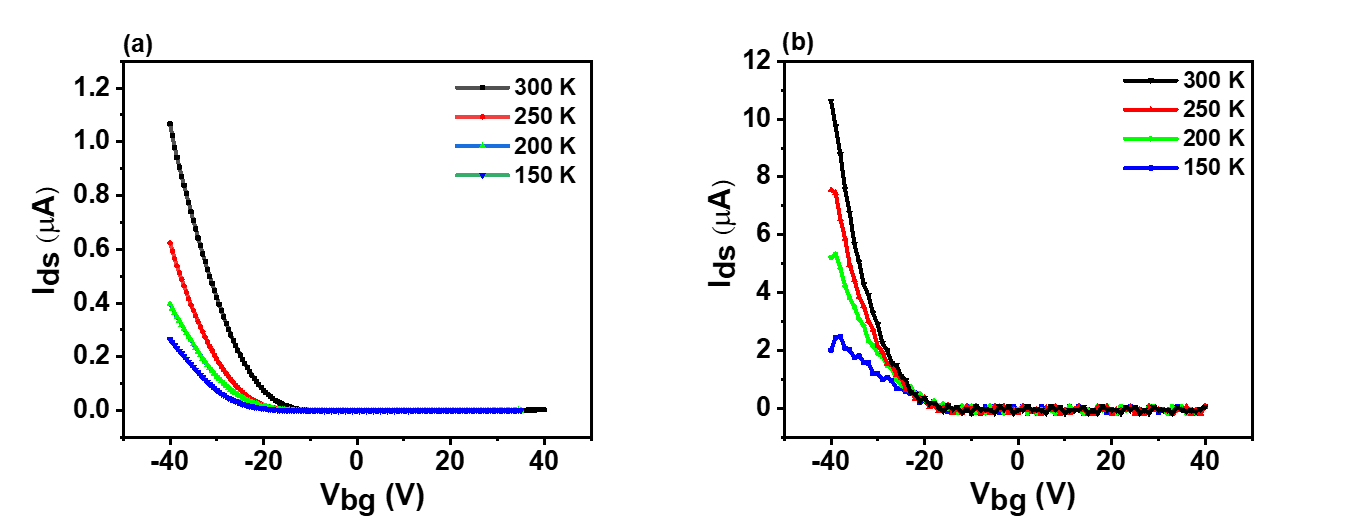


**Figure S2.** Temperature dependent transfer characteristics with (a) Cr/Au and (b) metal contacts.


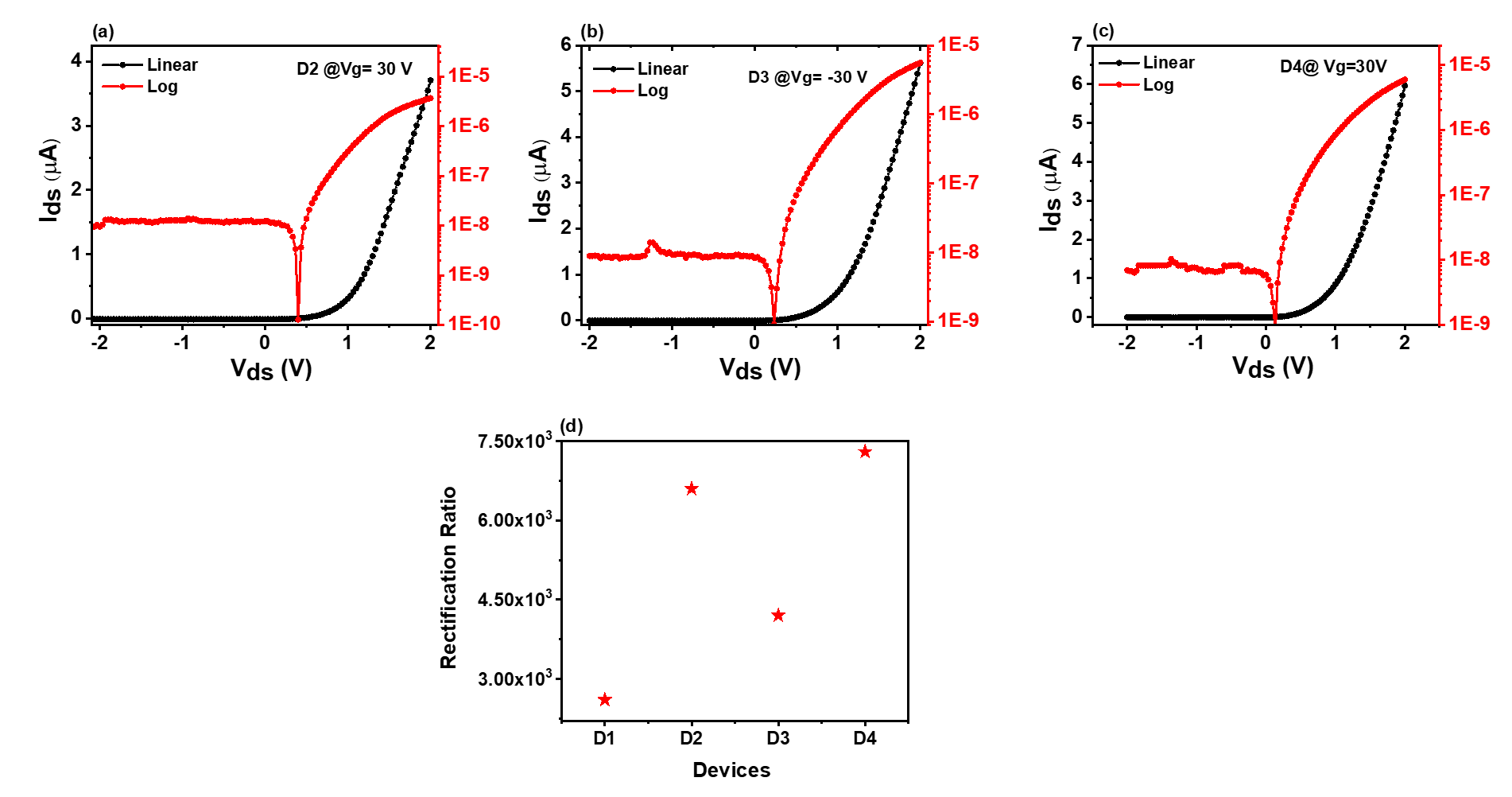


**Figure S3.** Repeatability of I*-V* characteristics *of* p-GeSe Schottky junction between Cr/Au-Pd/Au contacts at V_bg_= -30V. (a)Device 2 (b)Device 3 (c)Device 4. And (d) corresponding rectification ratio of devices.


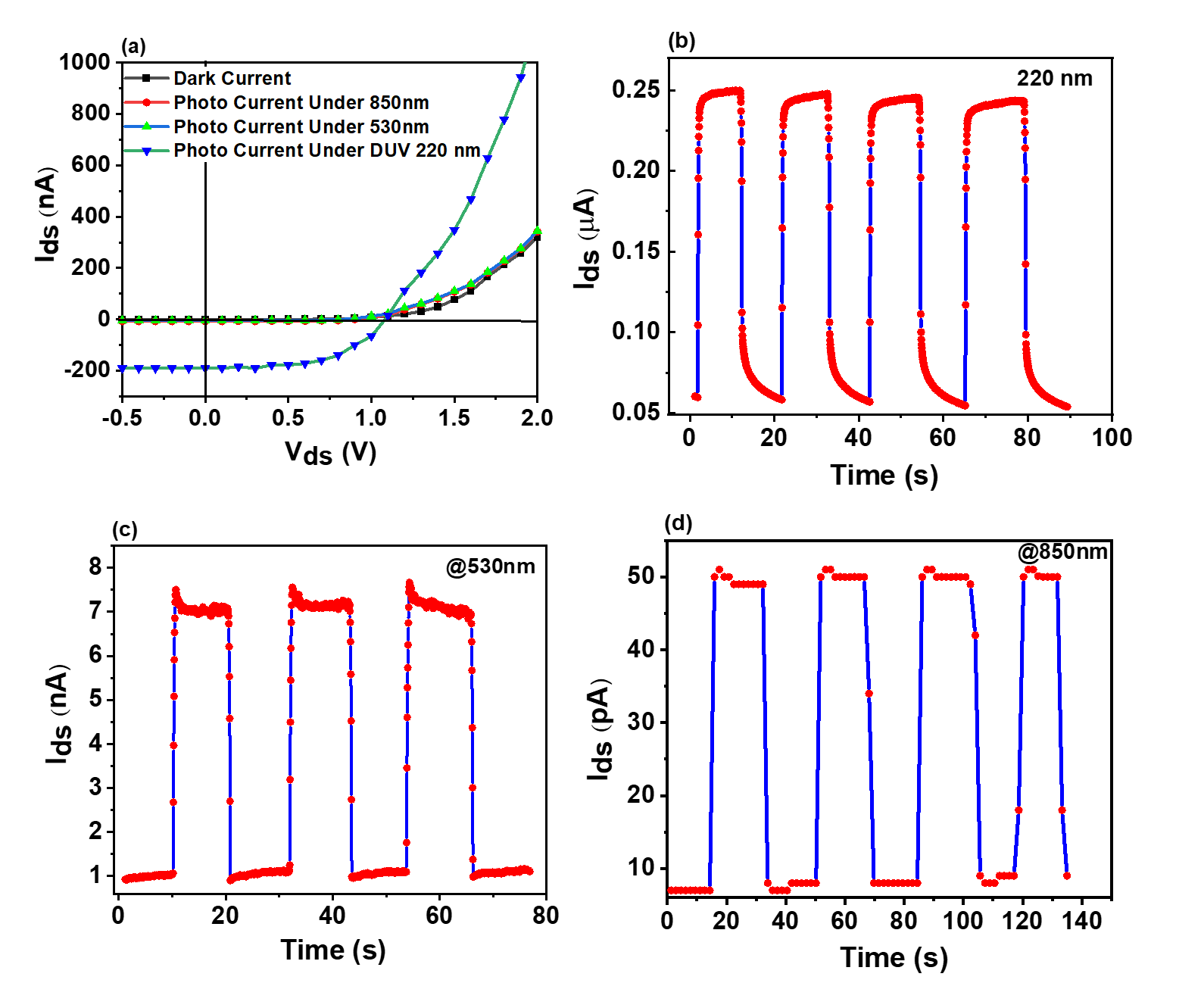


**Figure S4.** (a) wavelength dependent *I-V* characteristics of p-GeSe Schottky junction. Transient photoresponse of p-GeSe Schottky junction under illuminations with laser light at V_ds_ = 0V (b) with 850 nm. (c) with 530 nm and (d) with 850 nm.


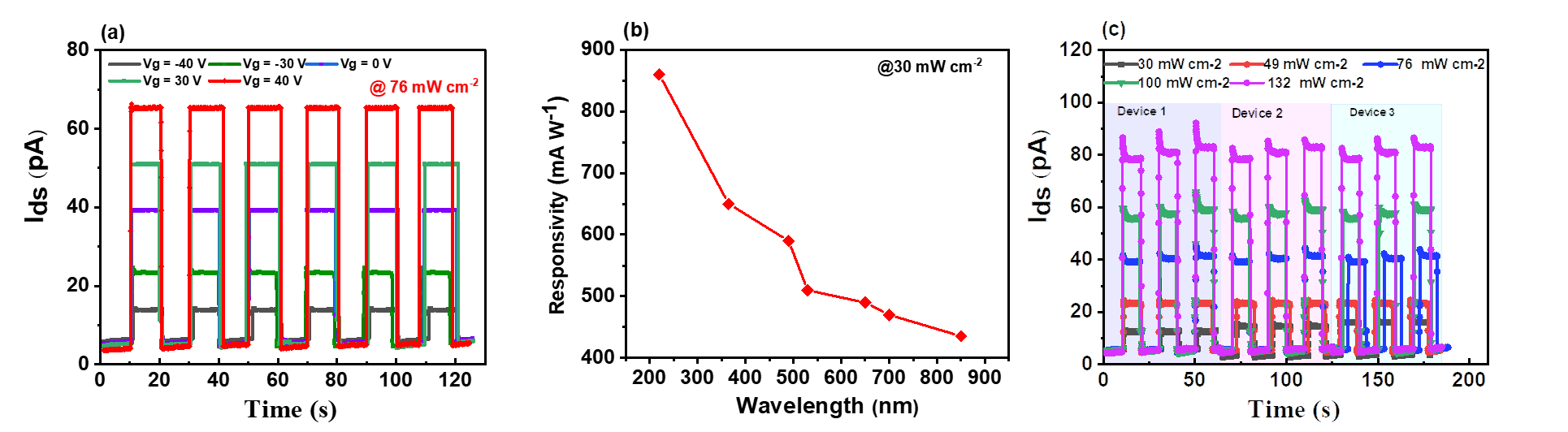


**Figure S5** (a) Gate tunable transient photoresponse of p-GeSe Schottky junction *V_g_* -40 V to +40 V with constant illumination power of 76 mW cm^-2^. (b) spectral photoresponse of p-GeSe Schottky junction ranging from 220 nm to 850 nm with constant illuminating power of 30 mW cm^-2^was characterized. Shown in Figure S 5b,we realized that the sharp increase of the spectral response on the short wavelength side is incontestably due to rapid increase of the photon’s energy absorbed by the device ,attributed to more electrons and holes generation under larger photons energy.(c) Transient photoresponse of different devices un various power illumination.

.
